# Differential gene expression in two consecutive pregnancies between same sex siblings and implications on maternal constraint

**DOI:** 10.1038/s41598-024-54724-3

**Published:** 2024-02-20

**Authors:** Theodora Kunovac Kallak, Solveig Serapio, Nadja Visser, Susanne Lager, Alkistis Skalkidou, Fredrik Ahlsson

**Affiliations:** Department of Women’s and Children’s Health, Uppsala University, 751 85 Uppsala, Sweden

**Keywords:** Computational biology and bioinformatics, Developmental biology, Genetics, Biomarkers, Diseases, Medical research

## Abstract

The objective of this study was to investigate how placental gene expression differs in two consecutive pregnancies in same sex siblings, and its possible association with the “maternal constraint” hypothesis. Material was gathered from the BASIC study (Biological, Affect, Stress, Imaging, and Cognition in Pregnancy and the Puerperium), a population based prospective study that was started in 2009 in Uppsala. Over 900 specimens of placenta biopsies were collected and out of these 10 women gave birth twice, to the same sex child, and were included in this study. The total RNA was isolated and prepared from frozen villous tissue from the placenta and further analyzed by use of Ion AmpliSeq Human Transcriptome Gene Expression kit. A total of 234 genes differed significantly between the first and second pregnancy placentas, when adjusting for delivery mode, maternal BMI and gestational age. Of special interest was the down-regulated group of genes in the second pregnancy. Exemplified by Pentraxin 3, SRY-Box Transcription Factor 9, and Serum Amyloid A1, which all were associated with biological processes involved in the immune system and inflammation. Further, protein–protein interaction analysis visualized them as hub genes interacting with several of the other differentially expressed genes. How these altered gene expressions affect maternal constraint during pregnancy needs further validation in lager study cohorts and also future validation in functional assays.

## Introduction

The prevalence of obesity among children, adolescents ,and adults has developed into one of the most serious public health concerns in the twenty-first century^1^. The prevalence of obesity has increased during the last decade, in the U.S it is estimated that approximately one out of three adults are obese with associated conditions such as cardiovascular disease, type 2 diabetes, hypertension, dyslipidemias, osteoarthritis, and certain cancers^2,3^. Women of reproductive age do not escape this epidemic^4,5^. It is well known that obesity in pregnancy is linked to several obstetrical complications both for the mother and the infant. It may increase the offspring’s risk for disease later in life, such as obesity, diabetes, cardiovascular problems, and certain malignancies. In addition, it is a burden on the health care resources^6–8^.

Previous epidemiological studies have shown a difference in phenotypic patterns between the first born compared to the second born child. Later in life, the first-born child is usually taller, heavier and has higher BMI than later-born children^9,10^. There are several hypotheses to why first born children have a greater BMI compared to their second born siblings, but the exact cause or mechanism is still unclear^11^.

Our group has previously shown that the higher the maternal BMI, the higher were the risks of developing overweight and obesity among their first-born daughters^12^. In addition, we have previously shown a higher risk for first-born females to develop obesity and overweight compared to the second born female siblings^13^. One possible reason for the increased risk of developing obesity in the firstborn female is the “maternal constraint” hypothesis, that refers to a weakly defined process by which maternal and placental factors limit fetal growth^13,14^. Although the exact mechanism of the constrain process is unclear, it occurs during all pregnancies. However, its more pronounced in e.g. twin pregnancies or in pregnant females who are of young age, short stature and in primiparas^14^.

We have previously shown that parity is a predictor of maternal leptin levels and gestational weight gain (GWG) during pregnancy, and also standardized birth weight of the child^15^. Further, we have also shown that parity can predict maternal testosterone levels, overall suggesting differential function of placenta between first and second pregnancy^16^. These findings thus suggest differential placental function associated with birth order. To the best of our knowledge, there is a gap of knowledge with regard to differential placental gene expression between two consecutive pregnancies.

Altered gene expression between consecutive pregnancies may be of importance for further understanding of fetal programming with regard to maternal constraint considerations. Therefore, the aim of this study was to investigate and compare gene expression in placenta from paired samples taken during the first and the second same-fetal-sex pregnancies.

## Material and methods

### Study population

Material has been gathered from the BASIC study (Biological, Affect, Stress, Imaging, and Cognition in Pregnancy and the Puerperium), that was started in 2009. The BASIC study is a population based prospective study, conducted by the Department of Women’s and Children’s Health at Uppsala University Hospital. The study includes information about pregnant women and their children. All pregnant women in Uppsala County, over 18 years of age, who speaks Swedish and were scheduled for a routine ultrasound at Uppsala University Hospital between 2009 and 2019, were invited to participate in the BASIC study. All participants gave informed consent and the study was approved by the Regional Ethical Review Board, Uppsala, Sweden, now called The Swedish Ethical Review Authority, registration number 2009/171 and 2012/308 and all procedures were according to approved study plan.

Women diagnosed with a pathological pregnancy (malformations leading to termination of pregnancy or miscarriage), with blood-borne disease, or with protected personal data were excluded^17^. Tissue biopsies and blood samples were collected at delivery. Within the study, different questionnaires were used to collect information regarding disease history, and background and demographical characteristics, such as BMI. Information was also gathered from antenatal care records of pregnant women enrolled in the maternal health care system in early pregnancy. Throughout the study, 900 specimens of placenta biopsies have been collected between 2010 and 2013. Out of these, 11 belonged to women who gave birth twice, to a same sex child, and these were included in this study.

### RNA isolation and sequencing

Placenta biopsies from the BASIC study cohort, were collected and processed directly after delivery. Two basal-plate biopsy samples from the maternal–fetal interface, approximately one centimeter in thickness were excised from the central part of the placenta containing both the maternal decidua basalis and fetal villous tissue. Affected areas in the placental tissues were avoided, such as blood clots. The tissue samples were carefully rinsed with sterile phosphate-buffered saline (PBS) and put on dry ice within 30 min after delivery. The samples were stored at − 70 °C until further processing^18^. The smaller biopsies used for RNA extraction were then sectioned out at the lab whilst keeping the biopsies on dry ice. At this moment, it was ensured that only villous tissue was taken and not decidua.

The total RNA was isolated and prepared from frozen villous tissues of the placenta. Total RNA was isolated using RNeasy mini kit (Qiagen, Hilden, Germany). RNA concentrations were measured with ND-1000spectrophotometer (NanoDrop Technologies, Wilmington, DE) and the quality of RNA was evaluated using the Agilent 2100 Bioanalyzer system (Agilent Technologies Inc, Palo Alto, California). and RNA integrity number (RIN) value > 4.0 were included. A total of 50 ng RNA was sequenced using Ion AmpliSeq Human Transcriptome Gene Expression kit on Ion 5SXL sequencer and reads aligned to hg19 AmpiSeq Transcriptome ERCC v1, by use of Torrent Suite v. 2.12.1 during 2020 at SciLifeLab, Uppsala, a national research infrastructure in Sweden.

### Statistical analysis demographic variables

The demographic variables were analyzed with paired t-test for normally distributed values and Wilcoxon signed ranked test for skewed distributed values. Frequencies were compared using Fisher's exact test. A p-value < 0.05 was considered significant. Statistical methods for RNA sequencing data are described below.

### Bioinformatic analysis of differentially expressed genes

Analysis was performed at Uppsala Multidisciplinary Center for Advanced Computational Science (UPPMAX) and bioinformatic tools were used to visualize affected pathways and biological systems that differ between two consecutive pregnancies. All analyses presented in this study were performed on the count data (expression_counts.tar.gz) provided by the sequencing facility. The datasets generated and analyzed during the current study are available in the SciLifeLab Data Repository SciLifeLab Data Repository, through the identifier 10.17044/scilifelab.24407893.

Gene filtering and data transformation was performed. Genes with very low expression were excluded (less than 3 samples with at least 10 counts). Gene expression data quality was assessed using variance mean dependency plots, heatmap of euclidean distances between samples and centered principal component analyses^19^. All differential expression analysis was performed on the raw count data from these 20 samples after filtering out low expression genes. DESeq2 version 1.32.0 was used for differential expression analyses^20^. Given that a filtering based on low expression was already performed, no additional independent filtering was done in DESeq2.

P-values were adjusted by using the Benjamini–Hochberg method^21^ and genes were considered statistically significant, if adjusted p-values was lower than 0.05. The first differential expression was paired by mother identifier with no other adjustment variables in model 1. The analysis was further adjusted by mode of delivery (categorized as spontaneous delivery vs. instrumental delivery which included deliveries with vacuum extraction, cesarean section or emergency cesarean-section.) in model 2^22^. Likelihood ratio tests were performed to assess whether interaction terms should be added in the models. These likelihood ratio tests compared models with and without interaction terms and 0.053% of the genes (8 genes) had adjusted p-values below 0.05 (p-value distribution close to uniform) so it was decided to not have interaction terms in the models. The last model 3 was adjusted for delivery mode, maternal BMI and gestational age at birth provided as continuous variables in days^22^. No likelihood ratio tests were performed on these fully adjusted models. For top significant genes of each analysis, normalized counts (variance stabilizing transformed data) were plotted for visualizing results.

A Gene Ontology (GO) enrichment analysis was performed on the unadjusted model 1 and fully adjusted model 3 analysis adjusting for delivery mode, maternal BMI, and gestational age. GO annotation was retrieved from the org.Hs.eg.db Bioconductor package version 3.13.0 (unique entrez gene identifiers from the org.Hs.egGO2ALLEGS object)^23^. Mapping between GO identifiers and GO terms was taken from GO.db version 3.13.0^24^. Among all GO terms, biological processes with at least 5 and at most 1000 genes were considered. Gene symbols were mapped to enter gene identifiers using the same version of org.Hs.eg.db^23^. Gene set enrichment analysis was performed using generally applicable gene-set enrichment (GAGE)^25^. For this, all genes were ranked based on their multiple testing adjusted p-values and direction of regulation so that most significantly up-regulated genes are on the top of the ranked list and most significant down-regulated at the bottom. Within GAGE, Wilcoxon-Mann–Whitney tests were chosen as enrichment tests for each direction (GO terms enriched in induced gene expression and suppressed gene expression). Enrichment tests p-values were also adjusted using the Benjamini–Hochberg method^21^. All analyses were done in R version 4.1.0 (2021–05-18)^26^. Gene lists (Supplementary table 1, 2) associated with each GO term were scanned for top 10 up-regulated (supplementary table 1) and down-regulated genes (supplementary table 2) identified by differential gene expression analysis. And also, for recurrent genes in GO terms top up-regulated or down-regulated genes.

### Protein–protein interaction analysis and transcription factor prediction

The free online tool Search Tool for the Retrieval of Interacting genes (STRING) (https://string-db.org/) was used for protein–protein interaction (PPI) analysis of the differentially expressed genes. The function “Proteins with values” were used with the log2 fold change values received in paired analysis described above, which was then analyzed by use of a normal geneset analysis. This was performed on down-regulated genes obtained with model 3. A medium required interaction score of 0.4 was used and no limitations on the number of interactions was set. Disconnected proteins was hidden in the exported network^27^. PPI analysis was used to identify hub genes.

The ChEA3 data base was used to identify transcription factors related to the differentially expressed genes. ChEA3 is a transcription factor enrichment tool designed to rank transcription factors in user-submitted gene sets. The mean rank method was used^28^. Target genes of selected transcription factors where then further investigated by use of information from https://genecards.org.

## Results

### Demographic characteristics

Characteristics of the study population are presented in Table 1. Data showed a strong significant difference between the two pregnancies regarding the maternal age which was expected due to the study design. No other maternal nor child characteristics were significant different between the first and second pregnancy on a group level.

**Table 1:** Demographic data.

Characteristics	First pregnancy (n = 10)	Second pregnancy (n = 10)	P value
Age	30.2 (3.795)	32.7 (3.68)	< 0.001
Maternal BMI	26.47 (4.13)	26.41 (4.3)	0.920
Birth weight	3627 (429.109)	3553 (387.959)	0.671
Sex of the child			
Boys	8 (80%)	8 (80%)	n/a
Girls	2 (20%)	2 (20%)	
GA (weeks)	39.9 (1.969)	39.5 (0.850)	0.591
GA (days)	282 (12.614)	277.1 (5.301)	0.162
Vaginal delivery	4 (40%)	8 (80%)	n/a
Instrumental delivery	6 (60%)	2 (20%)	n/a

Normally distributed data are given as mean (SD). Categorical values are given as percentage (%). Paired t-test was used for normally distributed values and Wilcoxon signed ranked test for skewed distributed values. Frequencies were compared using Fisher's exact test. A p-value < 0.05 was considered significant. Gestational age (GA). Non-applicable (n/a).

### Quality control of RNA and RNA sequencing

An RNA integrity number (RIN) value > 4.0 was used for RNA sequencing. Therefore, one sibling pair was excluded due to poor RNA quality. Resulting in a total of 10 paired siblings, two girl siblings and eight boy siblings included in the study. The > 4.0 cut-off was based on the results obtained by use of the Bioanalyzer system including the electrophoresis results. If there were two clear bands with peaks at 18S Fragment and 28 S Fragment, the sample was used, after a discussion with SciLifeLab core facility who performed the RNA sequencing^29^. There was no difference in RIN values between the first and second pregnancies (p-value = 0.905).

**Figure 1:**
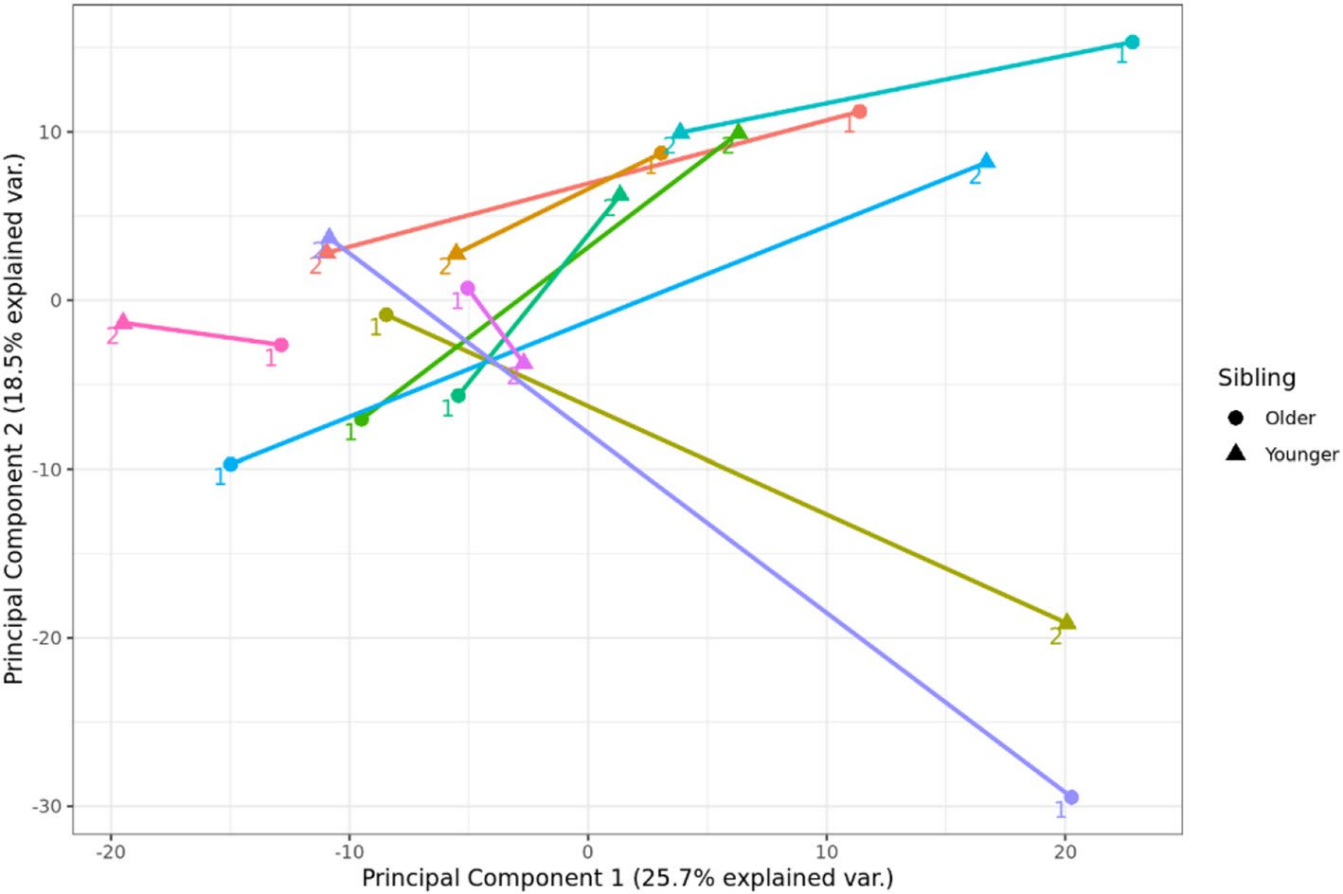
Centered principal component analysis of RNA sequencing data generated from 20 paired placental biopsies from ten consecutive pregnancies by use of Ion AmpliSeq Human Transcriptome Gene Expression kit of all samples are plotted in the first plane and colored based on sibling pairs and first (older) or second (younger) sibling.

RNA sequencing data quality was assessed. Variance mean dependency plots showed that variance stabilizing transformation (VST) corrects for the heteroskedasticity of the data whilst a log 2 transformation did not, presented in Supplementary Fig. 1. The heatmap of euclidean distance between samples did not show any specific batch effect caused by different sampling time for the two siblings in each sibling pair, presented in Supplementary Fig. 2. Global difference was also investigated by use of centered principal component analyses showing no global batch effect by sequencing batch Supplementary Fig. 3. Or any global gene between first and second born siblings presented in Fig. 1. An MA plot visualizing the log2 fold change in relation to variance stabilizing transformed normal counts of significant genes is presented in Supplementary Fig. 4.

### Differential gene expression in two consecutive pregnancies

**Figure 2:**
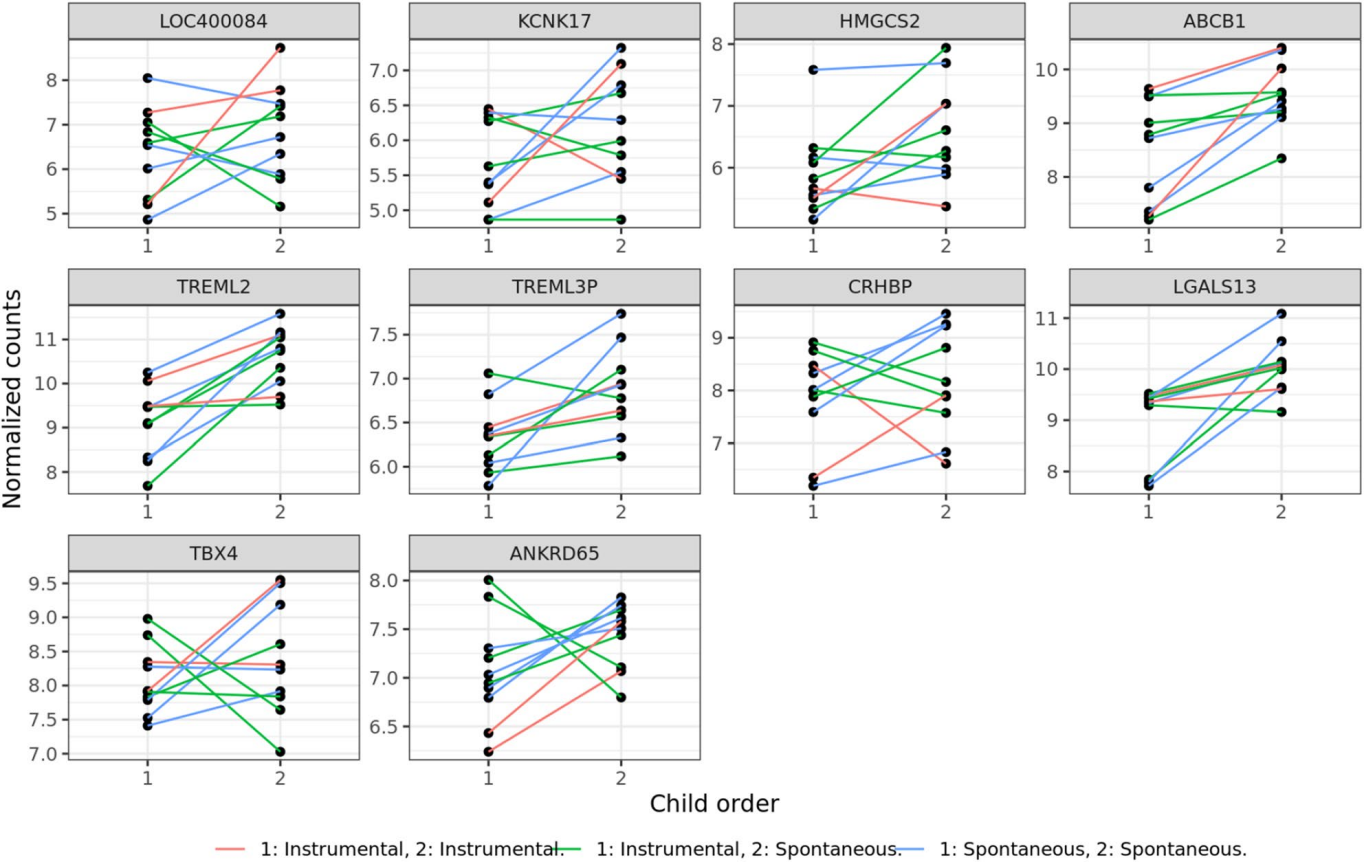
RNA sequencing data from 20 placental biopsies from ten consecutive pregnancies generated by use of Ion AmpliSeq Human Transcriptome Gene Expression kit. Here visualized as the top 10 up-regulated genes with the largest positive log2 fold change with adjusted p-value below 0.05 between the first and second pregnancy, adjusted for delivery mode, maternal BMI and gestational age. A line is plotted between siblings and this line is colored according to the modes of both deliveries.

**Figure 3:**
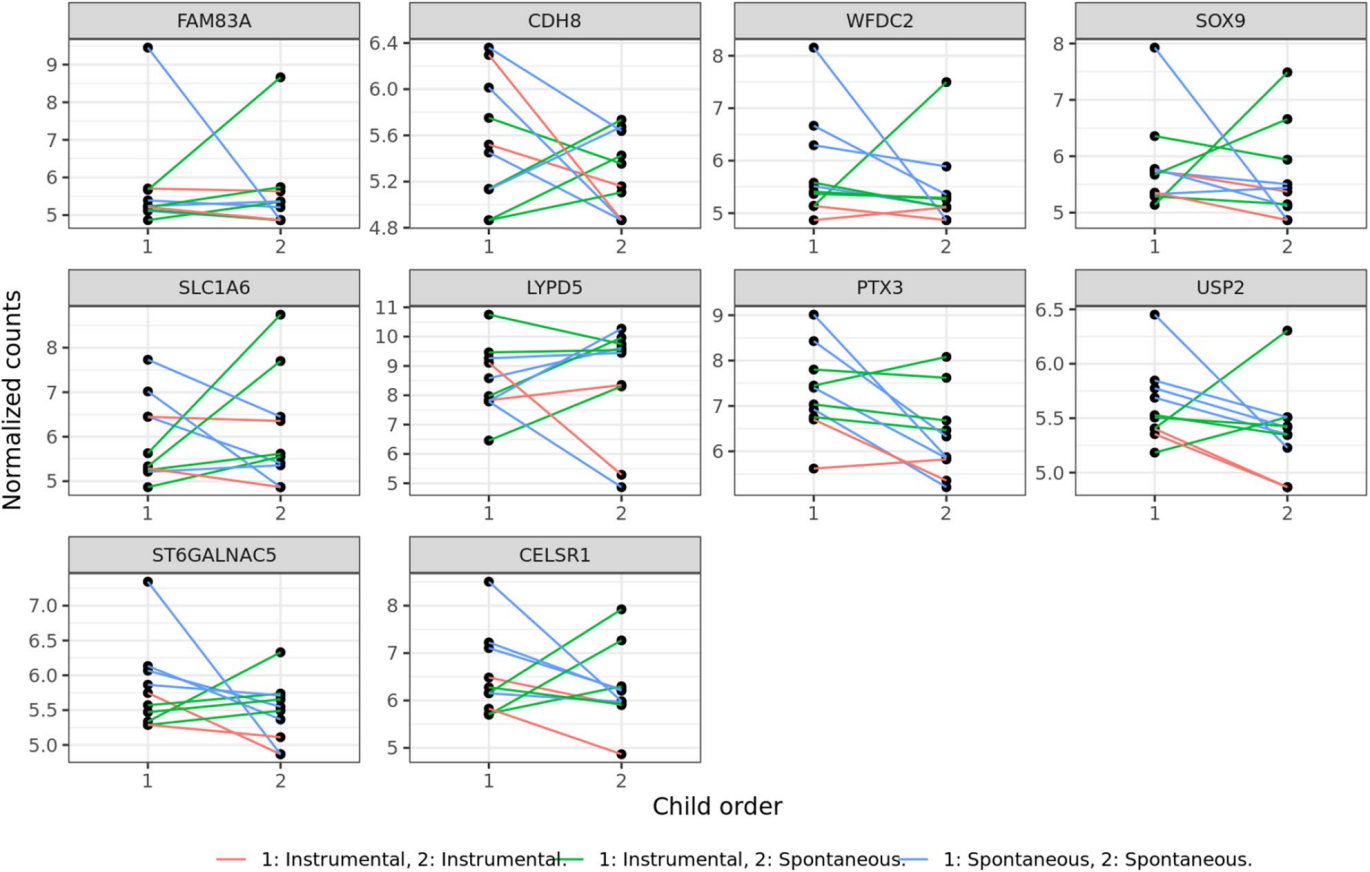
RNA sequencing data from 20 placental biopsies from ten consecutive pregnancies generated by use of Ion AmpliSeq Human Transcriptome Gene Expression kit. Here visualized as the top 10 down-regulated genes with the largest negative log2 fold change with adjusted p-value below 0.05 between the first and second pregnancy, adjusted for delivery mode, maternal BMI and gestational age. A line is plotted between siblings and this line is colored according to the modes of both deliveries.

In all comparisons, up-regulated genes should be interpreted as having a higher expression in the second pregnancy, while the down regulated genes as having a lower expression in the second pregnancy when looking at group level. Differential expression in the paired analysis in model 1 without any adjustments for covariates identified 35 genes that were significantly up-regulated and 85 genes that were significantly down-regulated. In model 2, when adjusted for delivery mode, there were 45 genes significantly up-regulated and 185 genes significantly down-regulated. When adjusting for delivery mode, maternal BMI and gestational days, know to affect placental gene expression, (model 3), 53 genes were significantly up-regulated while 181 genes were significantly down-regulated. The top ten up-regulated and down-regulated genes in model 3 are presented in Table 2 and Figs. 2 and 3 where, normalized counts (VST data) were plotted for visualizing results. The results of model 3 was used for further analysis by use of GO, PPI and transcription factors prediction.

**Table 2:** Top 10 up- and 10 down-regulated genes.

Gene title	Gene symbol	Log2 fold-change	P-value	Padj
	LOC400084	4.26E + 00	6.33E-04	4.51E-02
Potassium Two Pore Domain Channel Subfamily K Member 17	KCNK17	3.73E + 00	9.48E-05	1.12E-02
Hydroxymethylglutaryl-CoA Synthase, Mitochondria	HMGCS2	2.65E + 00	1.43E-06	4.83E-04
ATP Binding Cassette Subfamily B Member 1	ABCB1	2.00E + 00	1.54E-12	1.17E-08
Triggering Receptor Expressed On Myeloid Cells Like 2	TREML2	1.64E + 00	2.35E-05	4.02E-03
Triggering Receptor Expressed On Myeloid Cells Like 3	TREML3P	1.56E + 00	3.01E-04	2.77E-02
Corticotropin Releasing Hormone Binding Protein	CRHBP	1.55E + 00	5.45E-04	4.15E-02
Lectin, Galactoside-Binding, Soluble, 13/ Placental Protein 13	LGALS13	1.49E + 00	4.38E-05	6.24E-03
T-Box Transcription Factor 4	TBX4	1.44E + 00	3.39E-04	3.01E-02
Ankyrin Repeat Domain 65	ANKRD65	1.42E + 00	6.33E-05	8.31E-03
Family With Sequence Similarity 83 Member A	FAM83A	− 5.08E + 00	2.59E-07	1.36E-04
Cadherin 8	CDH8	− 4.61E + 00	6.08E-04	4.41E-02
WAP Four-Disulfide Core Domain 2	WFDC2	− 4.46E + 00	2.65E-04	2.50E-02
SRY-Box Transcription Factor 9	SOX9	− 4.23E + 00	1.72E-04	1.80E-02
Solute Carrier Family 1 Member 6	SLC1A6	− 3.72E + 00	1.38E-05	2.73E-03
LY6/PLAUR Domain Containing 5	LYPD5	− 3.63E + 00	5.86E-04	4.33E-02
Pentraxin 3	PTX3	− 3.60E + 00	1.93E-10	3.26E-07
Ubiquitin Specific Peptidase 2	USP2	− 3.59E + 00	1.54E-04	1.65E-02
ST6 N-Acetylgalactosaminide Alpha-2,6-Sialyltransferase 5	ST6GALNAC5	− 3.54E + 00	2.27E-04	2.25E-02
Cadherin EGF LAG Seven-Pass G-Type Receptor 1	CELSR1	− 3.47E + 00	8.70E-11	1.66E-07

Results showed the top 10 up- and 10 down-regulated genes based on log2 fold change from RNA sequencing data including 20 placental biopsies from ten consecutive pregnancies generated by use of Ion AmpliSeq Human Transcriptome Gene Expression kit. Paired analysis was used to compare placental gene expression between first and second pregnancy. The table represents the fully adjusted model 3 including adjustments for delivery mode, maternal BMI, gestational age.

### Gene ontology

Functional analysis was performed with several methods and the results presented are based on GAGE^25^. In the GO enrichment analysis performed on results from model 3, all genes were ranked according to their significance levels in the differential expression analysis (and the direction of their change, up or down). GO terms with genes significantly enriched on the top (enriched in induced gene expression, i.e. upregulation) or bottom (enriched in suppressed gene expression, i.e. downregulation) are provided in Tables 3 and 4. GO terms associated with upregulation of genes included biological processes of importance for mitosis such as mitotic cell cycle processes and chromosomal segregation. One of the top-upregulated genes, ATP Binding Cassette Subfamily B Member 1 (ABCB1), was noted amongst the genes involved in mitotic cell cycle process, cell cycle phase transition and mitotic cell cycle phase transition. Whilst, down-regulated genes were involved in processes related to inflammation and infection such as regulation of cytokine production, inflammatory processes etc. Three of the significantly top down-regulated genes were recurrently found in the GO terms: Pentraxin 3 (PTX3) in inflammatory processes and external encapsulating structure organization; SRY- Box Transcription Factor 9 (SOX9) in cell–cell adhesion and external encapsulating structure organization; Serum Amyloid A1 (SSA1) in regulation of cytokine production, cytokine production, inflammatory response, and cell chemotaxis (supplementary table 2). Amongst the genes with highest fold change and present in identified GO terms, LEP was recurrent in 6 out of 10 GO terms for down-regulated genes, including regulation of cytokine production, regulation of cell activation, cytokine production, inflammatory responses, cell–cell adhesion, and regulation of immune effector process (supplementary table 2).

**Table 3:** Gene ontology down-regulated genes.

Pathway	Stat.mean	p.val	q.val	set.size
GO:1903047 Mitotic cell cycle process	6.06	8.58e-10	3.82e-06	757
GO:0007059 chromosome segregation	6.11	9.83e-10	3.82e-06	266
GO:0000819 Sister chromatid segregation	6.05	1.97e-09	5.11e-06	168
GO:0000070 mitotic sister chromatid segregation	5.79	9.21e-09	1.79e-05	144
GO: 0098813 nuclear chromosome segregation	5.46	4.21e-08	6.55e-05	208
GO: 0045333 cellular respiration	5.44	5.83e-08	7.5e-05	148
GO: 0044770 Cell cycle phase transition	5.31	6.76e-08	7.5e-05	545
GO:0006260 DNA replication	5.32	7.97e-08	7.74e-05	241
GO:0044772 mitotic cell cycle phase transition	5.25	9.01e-08	7.78e-05	514
GO:0140053 mitochondrial gene expression	5.33	1.02e-07	7.95e-05	147

Top Gene ontology (GO)O terms within terms (biological process only) enriched in induced up-regulated gene expression identified in. RNA sequencing data including 20 placental biopsies from ten consecutive pregnancies generated by use of Ion AmpliSeq Human Transcriptome Gene Expression kit. Paired analysis was used to compare placental gene expression between first and second pregnancy. The table shows enriched GO terms based on the the fully adjusted model 3 including adjustments for delivery mode, maternal BMI, and gestational age.

**Table 4:** Gene ontology down-regulated genes.

Pathway	Stat.mean	p.val	q.val	set.size
GO: 0001817 Regulation of cytokine production	− 5.14	1.67e-07	0.000757	545
GO: 0050865 Regulation of cell activation	− 5.07	2.4e-07	0.000757	434
GO: 0001816 Cytokine production	− 4.93	4.71e-07	0.000757	580
GO: 0006954 Inflammatory response	− 4.85	7.09e-07	0.000757	536
GO: 0060326 Cell chemotaxis	− 4.87	7.95e-07	0.000757	210
GO: 0009617 Response to bacterium	− 4.83	8.19e-07	0.000757	418
GO: 0002250 adaptive immune response	− 4.84	8.37e-07	0.000757	306
GO: 0098609 Cell–cell adhesion	− 4.81	8.5e-07	0.000757	664
GO: 0002697 Regulation of immune effector process	− 4.83	8.76e-07	0.000757	300
GO: 0045229 External encapsulating structure organization	− 4.73	1.4e-06	0.00109	319

Top Gene ontology (GO) terms within biological process enriched in down-regulated gene expression identified in RNA sequencing data including 20 placental biopsies from ten consecutive pregnancies generated by use of Ion AmpliSeq Human Transcriptome Gene Expression kit. Paired analysis was used to compare placental gene expression between first and second pregnancy. The table includes GO analysis performed on down-regulated genes in model 3 adjusted for delivery mode, maternal BMI, and gestational age.

### Protein–protein interaction analysis and transcription factor prediction

By use of the online tool STRING, the PPI amongst the down-regulated genes in model 3 was analyzed. A total of 178 nodes were identified and 319 number of edges. The expected number of edges was 104 and therefore the PPI analysis received an enrichment score of < 1.0e-16 meaning that the downregulated genes in model 3 have more interaction than would be expected from a random set of genes. The results are presented in supplementary Fig. 5. Identified hub genes, meaning genes connected to other genes in this network, included SOX9 interacting with 17 other proteins, SAA1 interacting with 10 other proteins, and PTX3 interacting with five other proteins.

The transcription factor enrichment analysis was used to investigate which transcription factors were predicted to regulate our differentially expressed genes and also to see if some of these were differentially expressed in this study. We identified 12 transcription factors differentially expressed amongst the down-regulated genes in model 3, presented in supplementary table 3. SOX9 was one of these 12 transcription factors. SOX9 has transcription factor binding sites in three of the other identified differentially expressed genes which were also transcription factors themselves, including KLF transcription Factor 11 (KLF11), FOS Like 2, AP-1 Transcription Factor Subunit (FOSL2), and BCL6 Transcription Repressor (BCL6).

## Discussion

This study was conducted to identify possible differences in placental gene expression between same sex siblings, in two consecutive pregnancies, in order to investigate possible mechanisms contributing to maternal constraint. Little is known regarding gene expression changes over pregnancy and to our knowledge even less is known about gene expression changes between two consecutive pregnancies.

There were 234 genes that differed significantly between the first and second pregnancy placentas. Up-regulated genes were involved in biological functions of importance for mitosis such as mitotic cell cycle processes, chromosomal segregation, important for cell proliferation. Of special interest is, however, the down regulated genes in the second pregnancy, associating with biological processes involved in the immune system and inflammation. This could suggest that the activation of the immune system may not be as strong and probably better calibrated during the second pregnancy compared to the first pregnancy. That in itself could lead to improved fetal growth. One previous study investigated gene expression changes over the course of a normal pregnancy and an increment of gene expression was seen in genes associated with oxygen transport and the immune system^30^ which agrees with our results in relation to immune system. Consistent with our study and other studies is that when investigating gene expression during normal pregnancies, there are similar biological pathways identified that reflects the actual physiological processes that occur during pregnancies such as immune response and inflammatory markers such as cytokines. To the best of our knowledge, no other study has investigated changes in placental gene expression between two consecutive pregnancies in healthy women carrying same sex siblings. Possible mechanisms for maternal constraint are most likely fine tuning of natural occurring processes, inflammation and immune response has been suggested as a possible mechanism^14^.Thus, this is in line with the findings in this study that genes in the second pregnancy were downregulated in relation to inflammation.

When manually inspecting the gene list of differential expressed genes , sorting the genes by Logfold2 change, we identified PTX3, SOX9, SSA1 and LEP genes presented in the GO term list. Interestingly, PTX3, SOX9 and SSA1 were also identified as hub genes in the PPI analysis. The down-regulation of PTX3, which associated with GO terms such as inflammatory processes and external encapsulating structure organization, further supports our hypothesis that inflammatory fine-tuning between pregnancies might be one expatiation associated with maternal constraint. PTX3 was also one hub gene identified in the PPI analysis, at protein level, interacting with five other differentially expressed genes in our data set. PTX3 is a member of the long pentraxin subfamily and has an important role in the innate immune system. It exerts both regulatory functions and is an indicator of inflammatory response in sepsis, tumor progression, autoimmune disorders and in preeclampsia. It Is also correlated with latter disease’s severity and may be thought of as a marker of placental dysfunction^31,32^. PTX3 has previously been investigated in placentas from pregnant women with late-onset preeclampsia and gestational diabetes mellitus. In that study placentas from women with pregnancies with and without preeclampsia and GDM were compared by use of RNA sequencing^33^. Interestingly, the study also investigated the association of maternal, delivery and fetal variables with placental gene expression, showing that several genes involved in inflammation and placentation (including PTX3) were linked with parity as in our study. The authors suggest that this may indicate that women giving birth to their second child may respond differently to the presence of invading trophoblast compared to first time pregnancy^33,34^. The findings from this study showing altered PTX3 gene expression associated with parity may suggest that PTX3 can be one of the regulators of maternal constraint and should be validated in further studies.

SOX9 gene, identified as a hub gene in the PPI and also transcription factor for other down-regulated genes in model 3, is a versatile transcription factor, that plays a role in tissue organogenesis, cell development and differentiation. The lack of this gene may lead to a skeletal malformation, called campomelic dysplasia. The gene is also involved in the sex reversal syndrome^35^. Studies highlight the importance of SOX9 gene in its role of maintaining the cell’s undifferentiated state, in particular cancer cells^36^. However, we couldn’t find any studies investigating its role in fetal growth or its effect on maternal constraint.

The third hub gene identified in the PPI analysis was SSA1. SSA1 is a major acute phase protein highly expressed in response to inflammation and tissue injury. SSA1 has been shown to be produced by placental trophoblasts and to increase at time of parturition^37^. It has also been found to be associated with preeclampsia and placental dysfunction^38^.

The LEP gene was another gene identified as frequently recurring in the GO terms associated with parity. Elevated levels of leptin during pregnancy is associated with maternal obesity, were as decreased levels of fetal plasma levels is seen in children with intrauterine growth restriction^39,40^. There are even studies indicating a correlation between cord blood leptin concentrations and the effect on birth weight, however these conclusions are contradictory in many studies and various results have been published^41^. In one of our previous studies we investigated leptin levels from pregnant women with different BMI and its possible association with birth weight, and found that there was no association between maternal leptin levels and birth weight. The child birthweight seemed to be dependent upon maternal height, BMI, GWG and parity but not on the leptin levels^15^. Altered leptin gene expression has been identified in placenta from children born large for gestational age^42^ which indicates a possible role for leptin in fetal growth in the placenta that is not detectable in maternal plasma during the second trimester. Leptin may in fact affect the placental nutrient transport, and indirectly affecting the fetal growth.

The results of this study should only be considered whilst also taking the study limitations into account. First of all, the maternal constraint hypothesis suggest that the second born sibling has a higher birth weight than the first-born sibling, which was not the case in our study. There was no significant difference in body weight between the siblings. The nature of the project, investigating gene expression from two consecutives pregnancies from the same women giving birth to a child of the same sex, limited the number of participants for this study. In addition, the fact that out of these 10 women whom gave birth to the same sex child, there were eight boy sibling pairs and two girl sibling pairs which is also a limitation. It would have been better to have a larger study population with heavier second born children, and also an equal distribution of both sexes in the sibling pairs in order to conduct sex specific analysis. One minor limitation is also the lack of knowledge if the father is the same between the two pregnancies, since the information is not available in Swedish medical records. Even if both parents are the same, the gene expression comparison between siblings is confounded by the fact that there will always be genetic differences between siblings.

Gene expression in the placenta differs throughout pregnancy and many studies reflect differences in expression of genes, depending on type of samples taken, including sampling sites and sex of the fetus. For instance, samples taken from different tissues show individual differences in gene expression^43^. One study compared gene expression in the placenta depending on the timing and the site of collected tissue biopsy and could identify significant changes in gene expression. A difference has also been seen due to the apoptotic features that occur in the placenta near birth and term of pregnancy^44^. This may also influence the results and biological processes affected when analyzing gene expression during pregnancy, depending on mode of delivery. Sex related gene expression may also vary, as showed in previous studies but may in fact be related to the different physiology of them. Placentas from female infants show higher placental gene expression levels of genes involved in immune regulation compared to placentas from male infants^43^. Thus, one of the strengths in this study is that we only included placenta samples from same sex infants during two consecutive pregnancies.

Further strengths of our study were that placental biopsies were processed directly after delivery. Two basal-plate biopsy samples were taken from the maternal–fetal side and excised from the most central part of the placenta. The procedure was carried out in standardized way and the samples were snap frozen on dry ice. Our analysis investigating batch effects based on sampling time did not show any overall effects on gene expression which is a strength of the study. An additional strength is the adjustment for possible effects of delivery and gestational age^22^ on placental gene expression in the analysis.

## Conclusion

Birth order has previously been shown to predict size at birth^15^. This study, which utilized a unique study cohort of placentas from two consecutive same sex siblings, found several differentially expressed genes. In the downregulated group of genes, many were related to the immune system and inflammation. Genes of particular interest are PTX3, SOX9 and SSA1, which could affect how the placenta regulated fetal growth in utero*.* Larger study cohorts are needed in future studies to be able to draw more firm conclusions regarding how gene expression during two consecutive pregnancies affect maternal constraint and if it has implications on fetal programming for future obesity.

### Supplementary Information

Supplementary Figures.

Supplementary Table 1.

Supplementary Table 2.

Supplementary Table 3.

## Data Availability

The metadata has been deposited in SciLifeLab Data Repository through the identifier 10.17044/scilifelab.24407893. Contact persons, Theodora Kunovac Kallak and Alkistis Skalkidou.
