# Supplementary material for: Differential gene expression in two consecutive pregnancies between same sex siblings and implications on maternal constraint
Source: Sci Rep. 2024 Feb 20;14:4210. doi: 10.1038/s41598-024-54724-3 (PMC10879170; doi:10.1038/s41598-024-54724-3)
Supplement: Supplementary file 1 — Supplementary Figures. [file 41598_2024_54724_MOESM1_ESM.docx]

**Supplementary figures.**


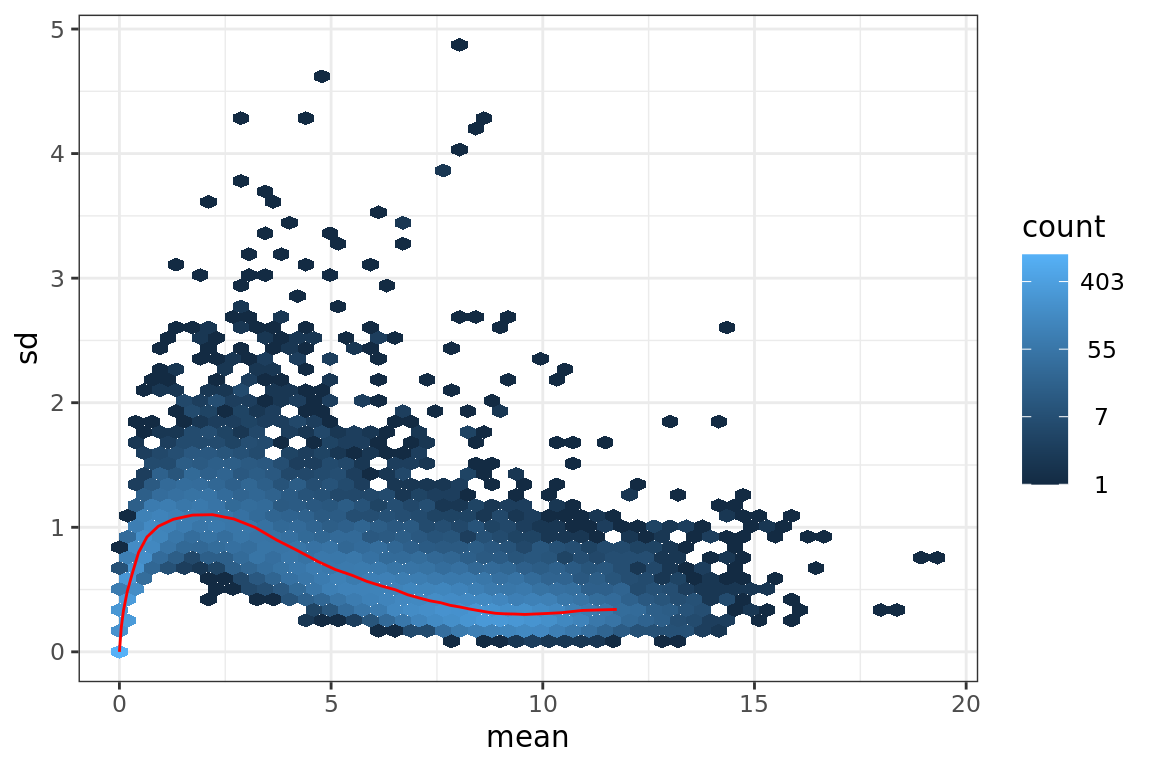
A)

B)


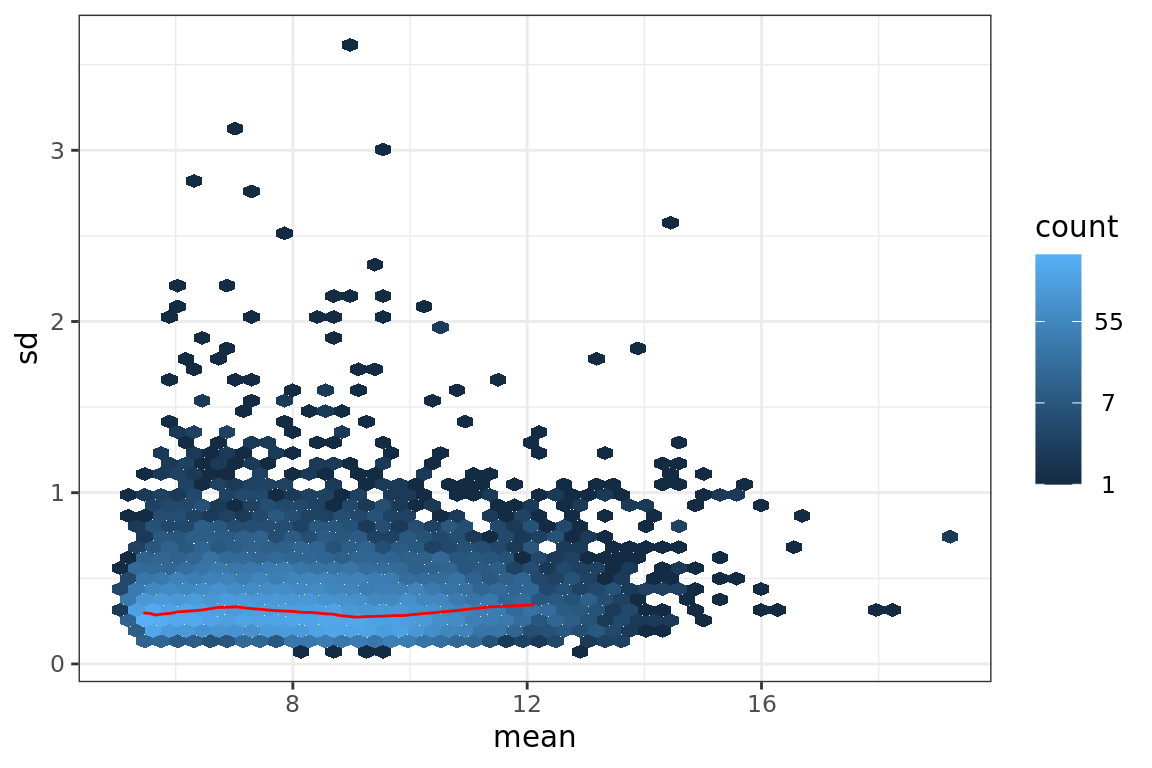


Supplementary figure 1.

Variance mean dependence plot presenting RNA sequencing data from 20 placental biopsies from ten consecutive pregnancies generated by use of Ion AmpliSeq Human Transcriptome Gene Expression kit with A) log2 transformed counts or B) variance stabilizing transformation (VST) normalized counts after filtering out low expression genes.


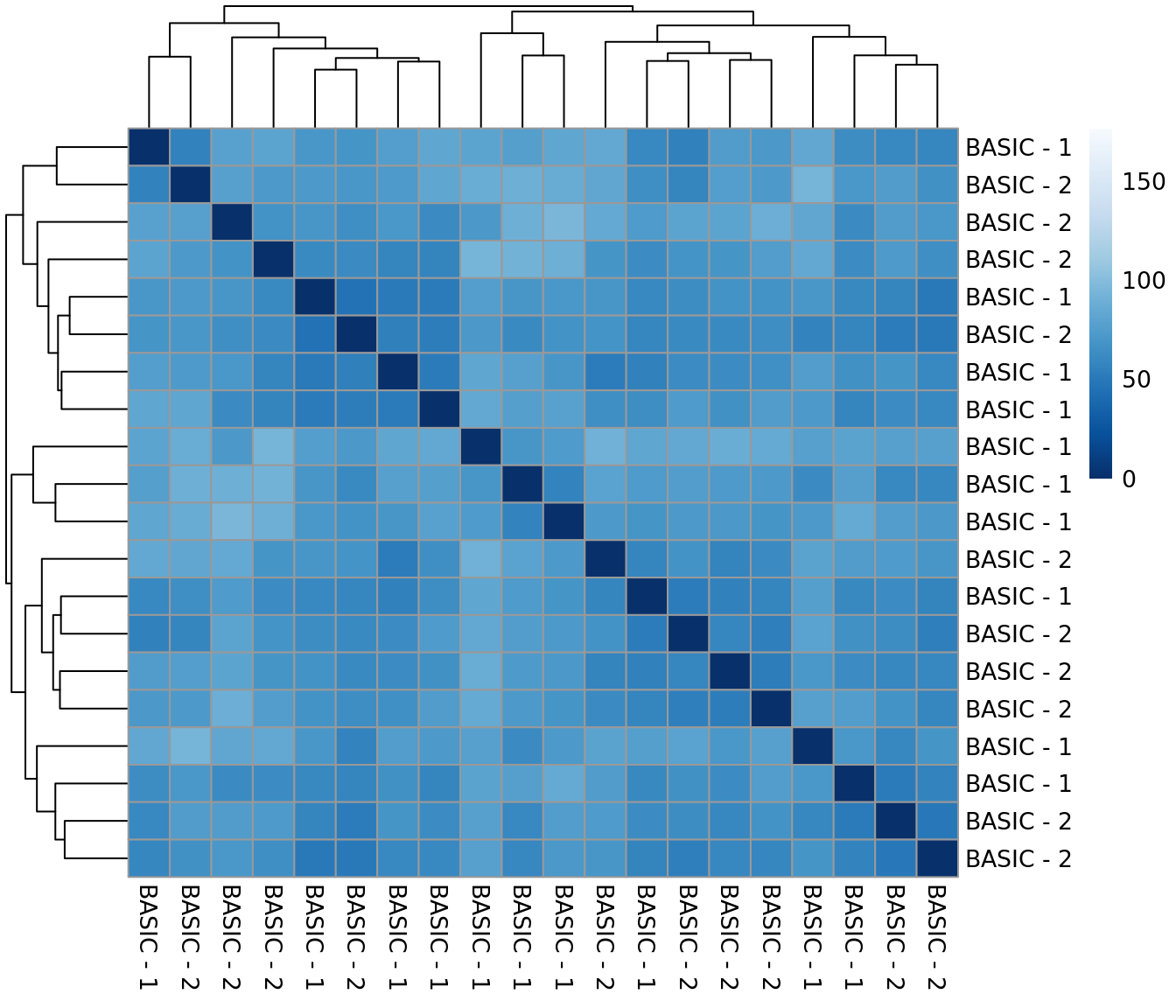


Supplementary figure 2.

Heatmap of euclidean distances between RNA sequencing results from 20 paired placental biopsies from ten consecutive pregnancies generated by use of Ion AmpliSeq Human Transcriptome Gene Expression kit. The color scale was chosen to cover the range of distances encountered in the dataset. Pairs of samples showing high gene expression similarities have low distance values (dark blue) and inversely meaning that samples diverting from the rest of the samples will have a light blue color.


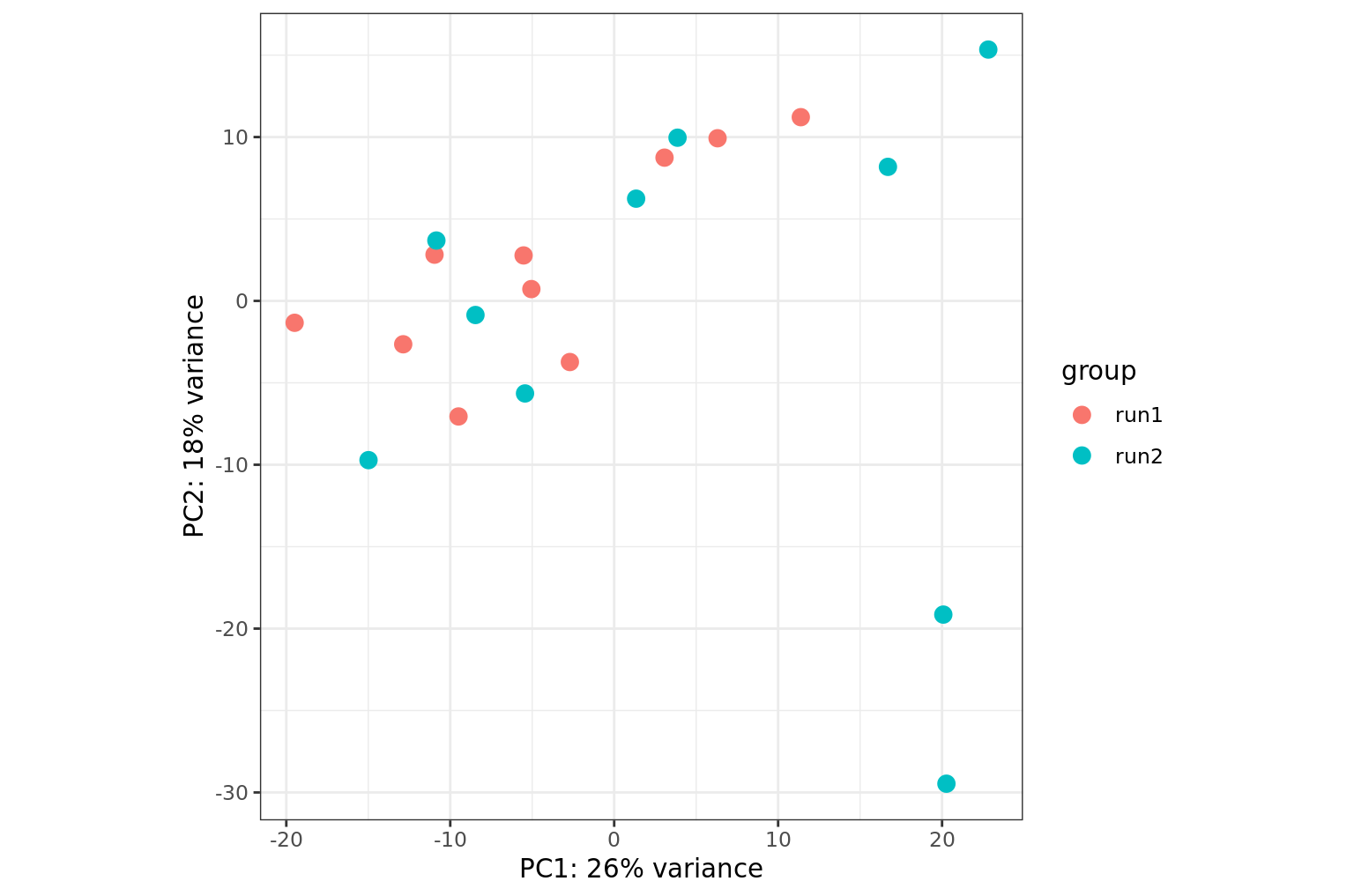


Supplementary figure 3.

Centered principal component analysis of RNA sequencing data generated from 20 paired placental biopsies from ten consecutive pregnancies by use of Ion AmpliSeq Human Transcriptome Gene Expression kit of All samples are plotted in the first plane and colored by sequencing run.


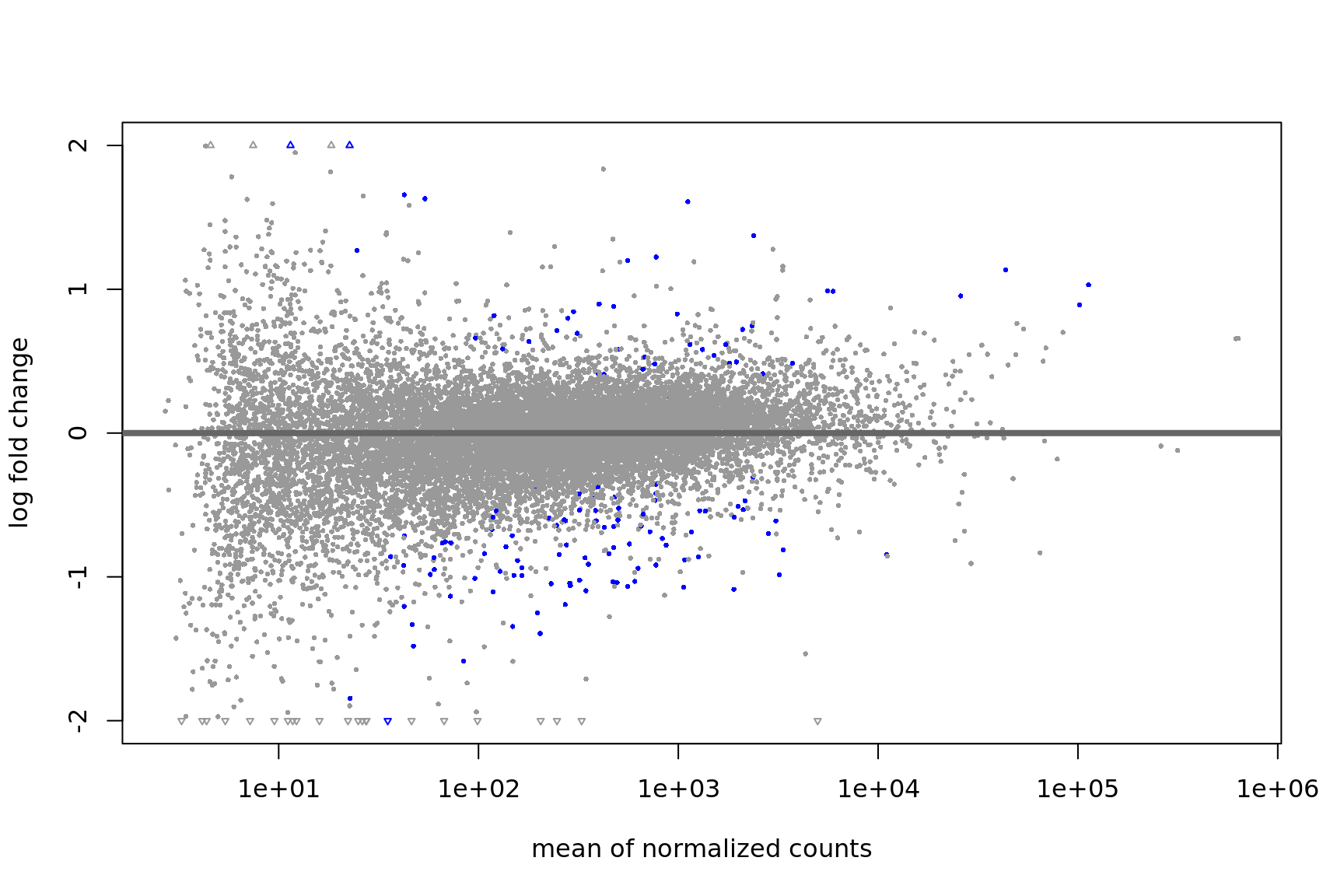


Supplementary figure 4.

MA plot of unadjusted RNA sequencing data generated from 20 paired placental biopsies from ten consecutive pregnancies by use of Ion AmpliSeq Human Transcriptome Gene Expression kit. The plot visualizes the log2 fold change in relation to variance stabilizing transformed normal counts, of significant genes which are colored in blue in relation to other genes expressed.

*
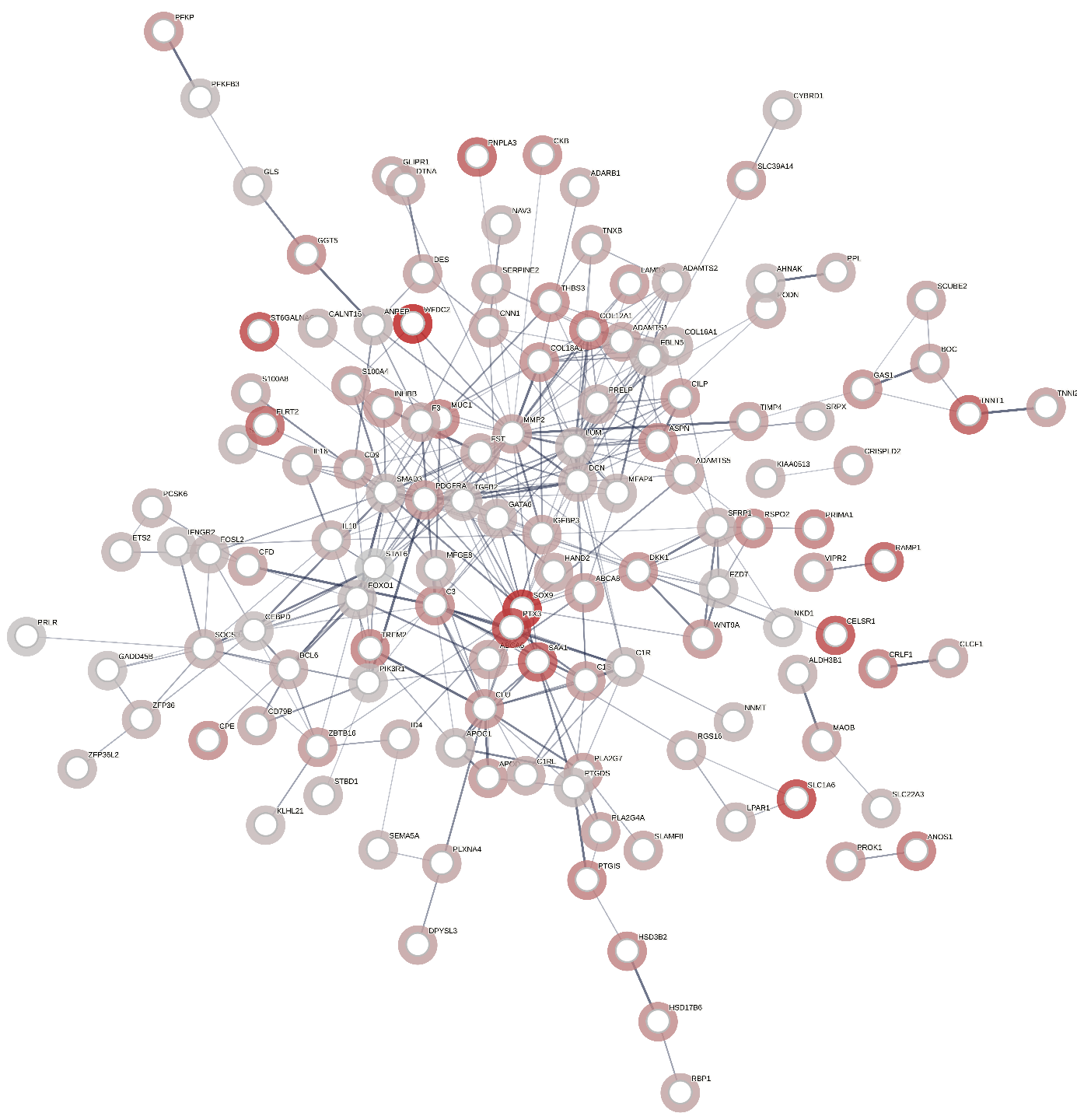
*

Supplementary figure 5.

The free online tool Search Tool for the Retrieval of Interacting genes (STRING) ([*https://string-db.org/*](https://string-db.org/)) was used for protein-protein interaction (PPI) analysis of the differentially expressed genes in RNA sequencing data generated from 20 paired placental biopsies from ten consecutive pregnancies by use of Ion AmpliSeq Human Transcriptome Gene Expression kit. The function “Proteins with values” was used with the log2 fold change values which as then analyzed by use of normal geneset analysis. This was performed on down-regulated genes obtained with model 3, paired group comparisons between first and second pregnancy placental gene expression adjusted for delivery mode, maternal BMI and gestational age at birth provided as continuous variables in days.
